# ITIS, a bioinformatics tool for accurate identification of transposon insertion sites using next-generation sequencing data

**DOI:** 10.1186/s12859-015-0507-2

**Published:** 2015-03-05

**Authors:** Chuan Jiang, Chao Chen, Ziyue Huang, Renyi Liu, Jerome Verdier

**Affiliations:** Shanghai Center for Plant Stress Biology, Shanghai Institutes for Biological Sciences, Chinese Academy of Sciences, Shanghai, 201602 China; University of Chinese Academy of Sciences, Beijing, 100039 China

**Keywords:** Transposable elements, Insertion sites, ITIS, Forward genetics, *Tnt1*, *Medicago truncatula*

## Abstract

**Background:**

Transposable elements constitute an important part of the genome and are essential in adaptive mechanisms. Transposition events associated with phenotypic changes occur naturally or are induced in insertional mutant populations. Transposon mutagenesis results in multiple random insertions and recovery of most/all the insertions is critical for forward genetics study. Using genome next-generation sequencing data and appropriate bioinformatics tool, it is plausible to accurately identify transposon insertion sites, which could provide candidate causal mutations for desired phenotypes for further functional validation.

**Results:**

We developed a novel bioinformatics tool, ITIS (Identification of Transposon Insertion Sites), for localizing transposon insertion sites within a genome. It takes next-generation genome re-sequencing data (NGS data), transposon sequence, and reference genome sequence as input, and generates a list of highly reliable candidate insertion sites as well as zygosity information of each insertion. Using a simulated dataset and a case study based on an insertional mutant line from *Medicago truncatula*, we showed that ITIS performed better in terms of sensitivity and specificity than other similar algorithms such as RelocaTE, RetroSeq, TEMP and TIF. With the case study data, we demonstrated the efficiency of ITIS by validating the presence and zygosity of predicted insertion sites of the *Tnt1* transposon within a complex plant system, *M. truncatula*.

**Conclusion:**

This study showed that ITIS is a robust and powerful tool for forward genetic studies in identifying transposable element insertions causing phenotypes. ITIS is suitable in various systems such as cell culture, bacteria, yeast, insect, mammal and plant.

**Electronic supplementary material:**

The online version of this article (doi:10.1186/s12859-015-0507-2) contains supplementary material, which is available to authorized users.

## Background

In the last decade, genome sequencing projects of economically or biologically important species have been a major driving force for biological research. After a genome is sequenced and annotated, functional characterization of genes that are important for development, cellular processes, or stress response becomes a major effort in the scientific community. Classically, gene function is determined using two opposite but complementary approaches: reverse genetics, which consists of deciphering the function of a gene by analyzing phenotypic effects of up- and down-regulations of the gene (i.e. from genotype to phenotype); and forward genetics, which consists of analyzing a phenotype (or trait) of a modified organism to identify gene(s) responsible for this particular phenotype (i.e. from phenotype to genotype).

Transposable elements (TEs) are mobile DNA sequences found in both prokaryote and eukaryote genomes. They usually represent a large proportion of the genome and under particular circumstances such as various stresses, some TEs become active and transpose themselves to other locations of the genome [1]. TEs are classified into two categories: Class I retrotransposons move following a “copy and paste” manner and class II DNA transposons following a “cut and paste” manner. Due to their mobility and depending on the location of their insertions, they may impact gene expression and gene function. Therefore, transposons are considered as a driving force of evolution and key players in adaptive mechanisms. In addition to specific endogenous transposons, exogenous transposable elements have been frequently used to develop numerous insertional mutant populations such as in mouse [1,2], yeast [3], nematode [4], fruit fly [5] and plants [6,7]. The development of insertional mutant populations in combination with forward genetic approaches have been widely used in functional genomics studies.

*Medicago truncatula* is a model plant to study legume biology and has been studied in different research areas such as seed biology [8,9], leaf development [10,11], symbiotic nitrogen fixation [12,13], secondary metabolite accumulation [14], response to stresses [15], and plant pathogens [16]. With the recent completion of a draft genome and its annotation [17,18], more than 60,000 gene loci have been identified and need to be functionally characterized. Large-scale mutant populations have been developed, including a *Tnt1* insertional mutant population [7], in order to accelerate functional genomics studies. The *Tnt1* mutant population was generated by transforming *M. truncatula* R108 with a well-studied Tobacco retrotransposon, called *Tnt1* [19]. *Tnt1* is a retrotransposon with a long open reading frame (ORF) in the center encoding enzymes that function in replication and transposition. The center region is flanked by 610 bp long terminal repeats (LTRs) in the same orientation (Additional file 1). During its transposition, *Tnt1* usually introduces a 5-bp target-site duplication (TSD) sequence on the two sides of the insertion site [20] (Additional file 1). The high efficiency of transposition of *Tnt1* during tissue culture enabled generation of a nearly saturated mutant population of *M. truncatula* genome [21], which has been widely used in reverse genetics approaches. In order to enhance the use of the *Tnt1* mutant population as a tool for forward genetics studies, efforts to identify flanking sequence tags (FST) of the *Tnt1* inserts have been made to ultimately identify the disrupted genes in different lines. High-throughput identification of FST sequences have been initiated using Thermal Asymmetric Interlaced (TAIL)-PCR-based approaches but technical limitations resulted in partial identification of *Tnt1* inserts.

Recent improvement of the throughput and decrease in experiment cost of next-generation sequencing (NGS) technologies permit the use of NGS to identify TE locations, FST sequences and ultimately disrupted genes at the genome-wide scale. Although several algorithms have been developed to identify TE insertion sites using whole genome re-sequencing data, most of them are not making full use of the information from short reads [22]. Whereas some of these algorithms detect insertions only by searching for cross reads pairs, where one read is aligned to the genome and its mate aligned to the inserted TE sequence [23-26], other algorithms only use clipped read pairs, in which a single read containing the TE sequence along with flanking reference sequence [27,28].

In this study, we present a newly developed bioinformatics pipeline, called ITIS (Identification of Transposon Insertion Sites), which was designed to identify flanking sequences of inserted transposable elements and ultimately genes disrupted from paired-end NGS data. Based on simulated and case study datasets, we demonstrated equal or superior sensitivity and specificity of our pipeline in comparison to other methods. By applying ITIS to a NGS dataset from a *M. truncatula* mutant line containing *Tnt1* insertions, we validated our method in a plant system by identifying and confirming TE insertion sites in the genome.

## Results and discussion

### Design and implementation of ITIS

**Figure 1:**
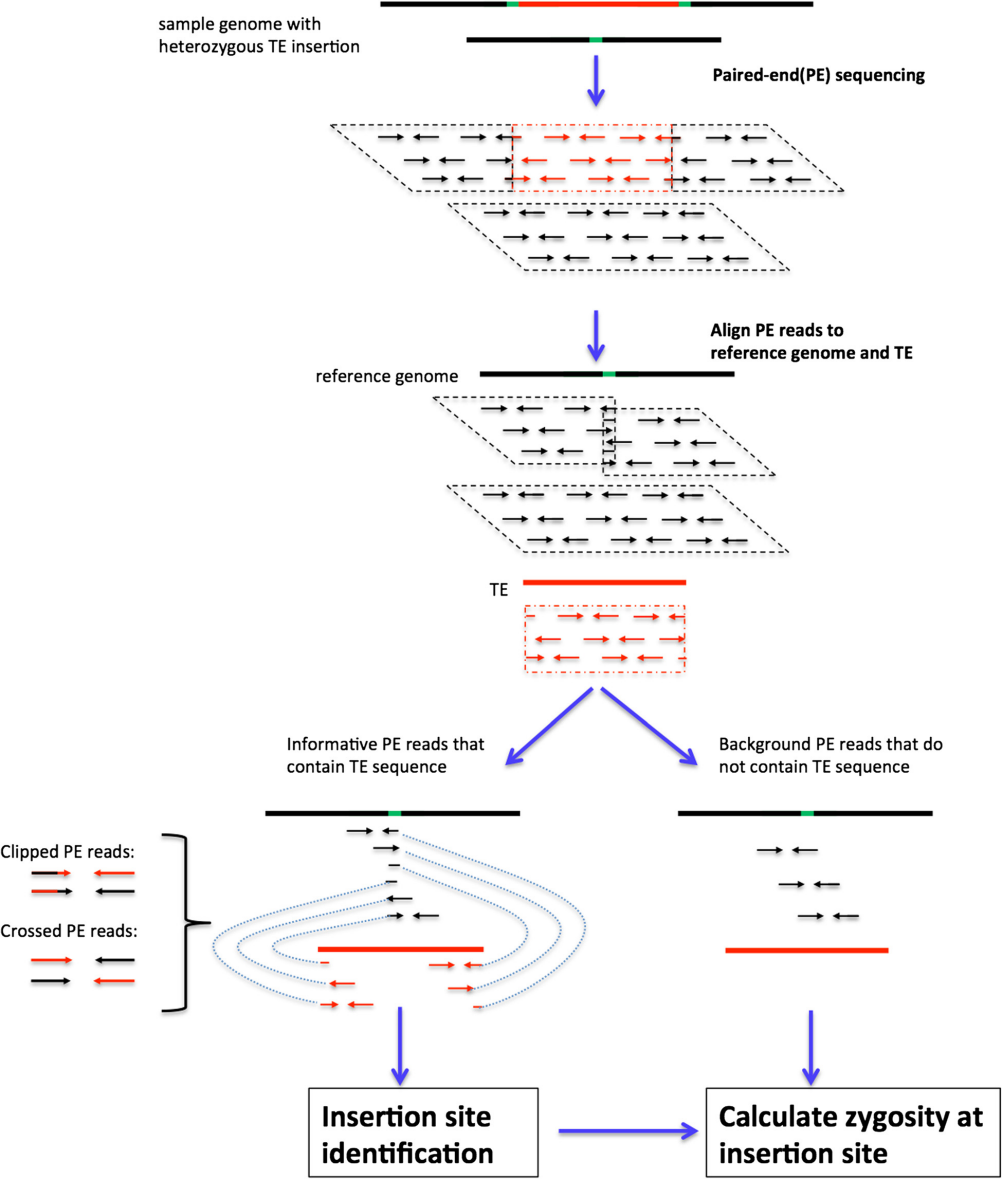
**Overall design and workflow of ITIS.** Black lines represent genome sequences, red lines represent TE sequences and green lines represent target site duplication sequences. Paired-end reads are denoted by arrow lines. The reads from the same genome region are framed by parallelograms.

ITIS requires three sequence files as input: (i) reference genome sequence, (ii) transposable element (TE) sequence, and (iii) paired-end (PE) short reads generated from the re-sequenced genome that contains novel TE insertions. The overall design of ITIS is illustrated in Figure 1. The first step of the ITIS pipeline is to map PE reads to the reference genome sequences and the transposable element sequence using short read alignment program BWA [29].

As second step, ITIS processes the alignment file and classifies PE reads into two categories. If both PE reads are mapped to the reference genome, but not the TE, they are regarded as background PE reads. Background PE reads do not provide information to determine insertion sites, but their numbers at the insertion loci are used to determine whether the insertion loci are heterozygous or homozygous. If at least one of the PE reads overlaps at least 20 bp with the insertion TE sequence, the PE reads are regarded as informative PE reads (Figure 1). Based on how the insertion junction is covered, the informative PE reads can be further classified into two types (Figure 1). The first type is cross PE reads, in which one end is completely mapped to the reference genome and the other end mapped to the insertion TE. The second type is clipped PE reads, in which at least one end covers both the reference genome and the insertion TE sequence. Clipped PE reads are used to determine the exact nucleotide position where the TE is inserted.

As third step, ITIS uses the genome coordinates of the informative PE reads to locate the cluster of informative reads. Each cluster indicates a candidate TE insertion site. In order to generate a list of insertion sites that are most likely genuine, ITIS uses a series of filters to discard possible false candidates by requiring that the average mapping quality of informative PE reads being at least 1 (to ensure that the insertion is not in repetitive regions), the average sequencing depth around the insertion being between 2 and 300, the number of supporting PE reads being at least 3, and both ends of the insertion must be supported by at least one read pair. Clipped PE reads are then used to determine the exact genomic location of the insertion.

Finally, for each detected insertion of TE, ITIS determines the likelihood of the insertion site being heterozygous or homozygous by using the number of informative PE reads (from the allele that does have the insertion) and the number of background PE reads (from the allele that does not have the insertion). ITIS was implemented in Perl and is freely available at http://bioinformatics.psc.ac.cn/software/ITIS. ITIS is a standalone software that runs in a Linux/Unix system from the command line.

### Evaluation of ITIS with a simulated dataset

**Figure 2:**
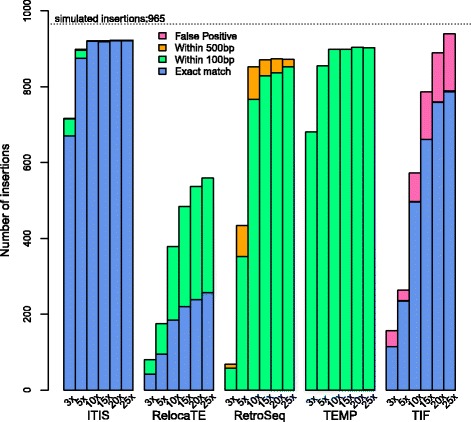
**Performance evaluation of ITIS and four other algorithms at different genome coverage using a simulated dataset.** The recovery rates of known insertions were compared among ITIS, RelocaTE, RetroSeq, TEMP and TIF. Identified insertions are considered false positive if their distance from the known insertion site exceeds 500 bp.

To evaluate the effectiveness and accuracy of the ITIS pipeline, we created a simulated benchmark dataset. An artificial transposon insertion mutant genome was created by *in silico* simulation with 965 *Tnt1* transposons (on average one insertion per 400 kb of genomic sequence) being randomly inserted into the *M. truncatula* A17 genome (version 4.0, [18]). Assuming that all insertion sites are heterozygous, equal amount of 100 bp PE reads were randomly extracted using the *pIRS* program [30] from the mutant genome and the reference genome to reach different coverages from 3X (around 12 millions reads), 5X (around 19 M reads), 10X (around 39 M reads), 15X (around 56 M reads), 20X (around 77 M reads) to 25X (around 96 M reads). These simulated reads were used as input to ITIS and the candidate insertion sites identified by the algorithm were compared to the known locations of the 965 insertions. By requiring that both ends of a TE were covered by at least one cross read pair or clipped read pair, we observed that at 3X genome coverage, 716 (74%) insertions could be correctly identified, among which 670 were assigned to the exact insertion positions, and 45 were located within 100 bp from the insertion sites (Figure 2). At 10X coverage, ITIS identified 921 (96%) insertions. However, the rate of identified insertions appeared to have reached plateau at 10X coverage and no additional insertion was recovered with higher genome coverage. After close examination, we found that the remaining undiscovered TE sites were inserted in repetitive sequence regions and, thus, could not be identified by ITIS with high confidence. These results demonstrated the high sensitivity of ITIS, which performed well with relatively low genome coverage (i.e. 5X) by identifying more than 90% of heterozygous insertions.

We also compared the performance of ITIS to four other similar algorithms: RelocaTE [24], RetroSeq [25], TEMP [26] and TIF [28] using the same simulated dataset as input. As shown in Figure 2, ITIS outperformed the other four algorithms in terms of sensitivity and specificity. ITIS was particularly effective when the genome coverage was relatively low (e.g. 3X and 5X) by accurately identifying the highest number of insertions. When the coverage was higher (e.g. >15X), TEMP and RetroSeq correctly identified similar number of insertions. However, RetroSeq and TEMP only allowed approximate locations of insertions within 100 or 500 bp to the insertion sites, whereas ITIS identified the exact insertion sites, showing better specificity. Even though TIF was able to correctly determine the exact insertion positions of the majority of the TE insertions, it was not as sensitive as ITIS at low coverage and displayed the highest rate of false positive insertions (Figure 2).

### Identification of *Tnt1* insertions from a *M. truncatula* mutant line

**Figure 3:**
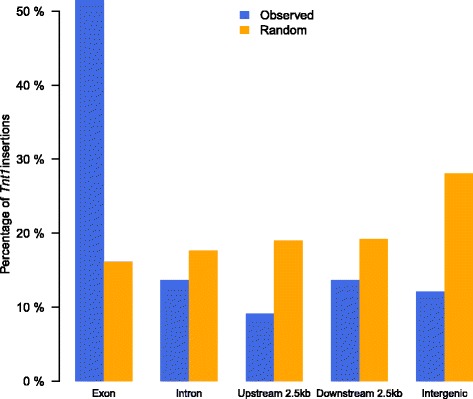
**Localization within the genome of** ***Tnt1*** **insertions in** ***M. truncatula*** **NF54.**

To further validate the effectiveness of ITIS, we applied ITIS to a *M. truncatula* mutant line from the *Tnt1* insertion mutant population. This mutant population is ideal for testing ITIS performance because of the two main challenges present in most of biological systems: (i) the presence of multiple *Tnt1* inserts within the genome at the homozygous and heterozygous states and (ii) this mutant population originated from a slightly different genotype (R108) than the reference genome A17, which was used by ITIS in our study. We selected mutant line, NF54, as case study, based on its interesting phenotypes to be studied further. We extracted DNA from leaf tissues and about 412 million clean PE reads were obtained, corresponding to 108X coverage over the 380 MB *M. truncatula* genome. We used ITIS to detect TE insertions in the genome and predict their zygosity based on allele frequencies (AFs). A total of 65 *Tnt1* insertions were identified in mutant line NF54 (Additional file 2). Among them, a total of 42 insertions (65%) were identified into genic regions, including 33 in exons and 9 in introns (Figure 3). The preference for insertions in genic regions was statistically significant, comparing to 10,000 simulated runs of random TE insertions (Fisher’s exact test; p-value < 4e-5, Figure 3). This result is consistent with previous reports in *M. truncatula*, potato and soybean [19,31,32], which showed that *Tnt1* insertion events preferentially occur in genic regions. 61 out of 65 insertions could be assigned to exact chromosomal positions. We further determined whether these insertion sites were in heterozygous or homozygous state. If a *Tnt1* insertion is in heterozygous state, the ratio of supportive PE reads (which cover two ends of the insertion) to background PE reads is 2:1 in theory. By the binomial test, we found that 16 out of 63 insertions were most likely homozygous (p-value < 0.05).

**Figure 4:**
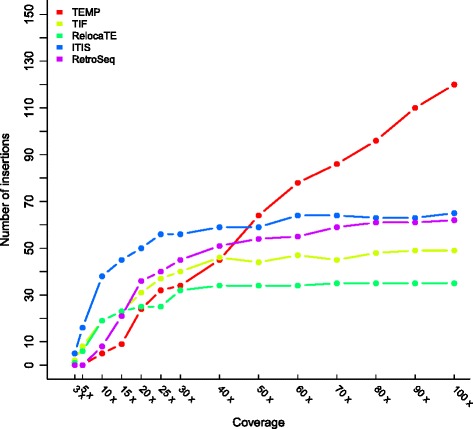
**Performance comparison of ITIS and four other algorithms at different genome coverage using NGS data from** ***M. truncatula*** **NF54.**

In order to evaluate how genome coverage affected the effectiveness of different programs, we randomly sampled PE reads from the sequence dataset generated from NF54 into subsamples representing 3X, 5X, 10X, 15X, 20X, 30X, 40X, … and 100X genome coverages and we examined the number of insertions that were identified using the four previously mentioned algorithms and ITIS. As shown in Figure 4, ITIS was the most effective at low genome coverage. Most of the insertions could be identified with 25X genome coverage, where the number of insertions reached a plateau. TIF, RetroSeq and RelocaTE recovered less insertions than ITIS at all coverage levels. TEMP identified more insertions than ITIS at high coverage (>50X). However, the number of identified insertions continued increasing until 100X coverage and was not consistent with other algorithm results, suggesting that additional identified insertion sites identified by TEMP might be false positives. To validate our hypothesis, we selected three out of the 70 additional inserts uniquely identified by TEMP, for PCR validation. None of the three putative insertions were confirmed by PCR amplification, which suggests that most, if not all, of these additional insertions identified by TEMP were false positive insertions (Additional file 3). To explain TEMP results, we inspected the 70 additional inserts uniquely identified by TEMP and found that all of them were located in repetitive regions, which might cause the unreliable TEMP identifications.

Comparing to the simulated dataset, ITIS required higher coverage to identify most of the insertions. It is most likely due to the fact that in the simulated dataset, reads were sampled from the reference genome (*M. truncatula* A17), but in the NF54 dataset, reads were generated from a closely related genotype (*M. truncatula* R108). Even considering the difficulty of read alignment between the reference genome (A17) and the PE reads from R108 due to genetic variations, ITIS showed better sensitivity and specificity than other algorithms.

### Validation of transposon insertions using database search and PCR-based approaches

To confirm ITIS identification in the mutant line NF54, we first compared ITIS results with the *Tnt1* insertions identified and stored in the *M. truncatula* mutant database. In parallel, we confirmed the presence of *Tnt1* inserts identified by ITIS using a PCR-based approach.

A few years ago, a large effort has been initiated to identify the FST sequences present in the *Tnt1* insertional mutant population [7]. To date, a tremendous work has allowed the identification of more than 157,000 high confidence (and more than 200,000 low confidence) FST sequences from the 22,000 *Tnt1* mutant lines using TAIL-PCR method. Positions and sequences of these FST sequences have been stored in the *M. truncatula* mutant database [33]. This web server represents a powerful tool in reverse genetics to identify mutant lines for specific candidate genes. However, its potential is limited for forward genetics study because of partial recovery of FST sequences present in the database due to the large size of the population (i.e. 22,000 lines) and limitations of the high-throughput TAIL-PCR method used. In the *M. truncatula* mutant database, 25 *Tnt1* flanking sequences were identified in NF54, however only 11 insertion sites were mapped in the genome. Out of these 11 putative insert positions identified by TAIL-PCR, ten were also identified by ITIS (Additional file 2). In order to validate insertions that were identified by ITIS, but not in the *M. truncatula* mutant database, we performed PCR-based validation of the presence and orientation of ten randomly selected putative insertions. We designed primers flanking the *Tnt1* insertion sites in order to test its presence or absence and primers on both sides of the *Tnt1* sequences (i.e. 5′ or 3′ side) to determine its orientation (5′ to 3′ or 3′ to 5′). Based on PCR results, both the presence and the orientation of all the different inserts tested were consistent with ITIS results (Additional files 2 and 4). Thus, in conclusion, the PCR-based approach validated ITIS results in term of presence, localization and orientation of inserts. In order to consolidate our experience, we sequenced a second *M. truncatula Tnt1* mutant line, NF200, at around 40X genome coverage (ie 165 M reads). Using these short read data, ITIS predicted the presence, location, orientation and zygosity of six *Tnt1* insertions in NF200 (Additional file 5). We performed PCR-based validation of five of them, and the presence, location, orientation and zygosity of all the inserts tested were consistent with ITIS results (Additional file 5).

## Conclusions

ITIS is a powerful bioinformatics tool suitable for identification of transposable elements for forward genetics. It may also serve as a valuable tool to identify phenotypic changes resulting from transposition events occurred at the species or sub-species level in evolutionary studies. It is suitable from low to high complexity genomes and performed well even using sequence dataset from genotypes that are closely related to the reference genome. By comparing with other similar algorithms, ITIS showed its superior or equal specificity, sensitivity and accuracy in a complex system. In conclusion, ITIS is a robust tool for identification of transposable elements in wide-ranging systems from cell to complex organisms such plant or mammal.

## Methods

### Deep sequencing data and reference sequences

Paired-end reads from genome of *M. truncatula* NF54 and NF200 were trimmed to discard low quality regions using SolexaQA (Q < 17) [34], and adapter sequences using cutadpt (v1.3) [35]. For NF54, 412 M clean reads were used to detect *Tnt1* insertion sites by ITIS and other algorithms. For NF200, 165 M clean reads were used to detect *Tnt1* insertion sites by ITIS. The draft genome sequence of *M. truncatula* was downloaded from the *M. truncatula* Genome Project V4.0 [18]. *Tnt1* sequence was downloaded from the NCBI database [GenBank: X13777]. Clean reads were mapped to reference genome with the parameter “–T 20” to ensure that at least 20 bp sequence of each read was properly mapped.

For simulation analysis, we randomly inserted 965 *Tnt1* sequences at different locations in the *M. truncatula* reference genome using an in-house Perl script. *pIRS* (v1.1.0) [30] was used with parameters “–l 100, -m 500 –v 50 –e 0.001 –a 0 –g 0” to generate simulated PE reads to obtain different genome coverages from 3X to 100X.

### Use of other algorithms to identify insertion sites

All other algorithms were set using default parameters, except for TEMP algorithm (insertions were filtered to be “1p1” and number of reads covering both 5′ and 3′ends of the TE was selected to be greater than 0).

### DNA extraction and whole genome sequencing

Plants of *M. truncatula* (R108) were grown in sterile soil under controlled conditions at 22°C with a 16 h photoperiod at 200 mmol.m^−2^.s^−1^ and 40% of relative humidity. Nuclear DNA was extracted and purified using Qiagen Plant DNeasy mini kit following manufacturer’s instructions and quantified using a NanoDrop 2000C spectrophotometer (Thermo Scientific). Two micrograms of DNA were used to create libraries using the standard Illumina TruSeq DNA sample preparation protocol. Whole genome sequencing was performed on an Illumina HiSeq2500 analyzer to generate paired-end reads of 100 bases (PE100).

### PCR-based validation

Presence or absence of inserts was checked by polymerase chain reaction (PCR) amplifications using the 2X Power *Taq* PCR mix (Bioteke Corp.) according to manufacturer’s instructions and using primers designed within the *Tnt1* sequence (5′ and 3′ sides of the *Tnt1* sequences) and on the flanking regions of the insertion sites. The complete list of primers is provided in the Additional file 6.

## Additional files

Additional file 1:
**Schematic diagram of a** ***Tnt1*** **insertion and the alignments of NGS reads.**
Additional file 2:
**List of the** ***Tnt1*** **insertional sites identified by ITIS in the** ***M. truncatula*** **mutant line NF54.** Table contains exact position, annotation of disrupted gene or intergenic region, gene ID, and zygosity. The middle column contains the PCR validation of the presence, location and the orientation of the insert. The right column contains the list of insertions identified in NF54 mutants using TAIL-PCR and available in the Medicago mutant database. Additional file 3:
**Results of PCR amplifications to verify the presence of** ***Tnt1*** **insertions uniquely identified by TEMP algorithm.** Reverse and forward primers were designed on the flanking sequences of the putative insertion sites. In combination with primers designed on both sides of the *Tnt1* sequences (5' and 3'), we did not observe any amplification, which suggest that these putative insertions identified by TEMP are false positives. Flanking sequences identified by TEMP are also provided. Additional file 4:
**Results of PCR amplifications to check presence and orientation of** ***Tnt1*** **insertions identified using ITIS algorithm and not present in the** ***M. truncatula*** **mutant database.** Reverse and forward primers were designed on the genomic sequences flanking the putative insertions. In combination with primers designed on both sides of the *Tnt1* sequences (5' and 3'), we validated the presence of all tested insertions. According to the combination of primers, we confirmed the orientation of the insertions revealed by ITIS. Additional file 5:
**List of the** ***Tnt1*** **insertional sites identified by ITIS in the** ***M. truncatula*** **mutant line 200.** Table contains exact position, annotation of disrupted gene or intergenic region, gene ID, and zygosity. The middle column contains the PCR validation of the presence, location, orientation and zygosity of the insert. Results of PCR amplifications to check presence, orientation and zygozity are also provided. Additional file 6:
**List of primers used for PCR validation of different insertion sites.**

## Abbreviations

PE: Paired-ends
TE: Transposable element
LTR: Long terminal repeats
ORF: Open reading frame
F: Allele frequency
NGS: Next generation sequencing
PCR: Polymerase chain reaction
FST: Flanking sequence tag
