# Supplementary material for: ITIS, a bioinformatics tool for accurate identification of transposon insertion sites using next-generation sequencing data
Source: BMC Bioinformatics. 2015 Mar 5;16(1):72. doi: 10.1186/s12859-015-0507-2 (PMC4351942; doi:10.1186/s12859-015-0507-2)
Supplement: Additional file 1: — Schematic diagram of a Tnt1 insertion and the alignments of NGS reads. [file 12859_2015_507_MOESM1_ESM.pptx]

## Slide 1
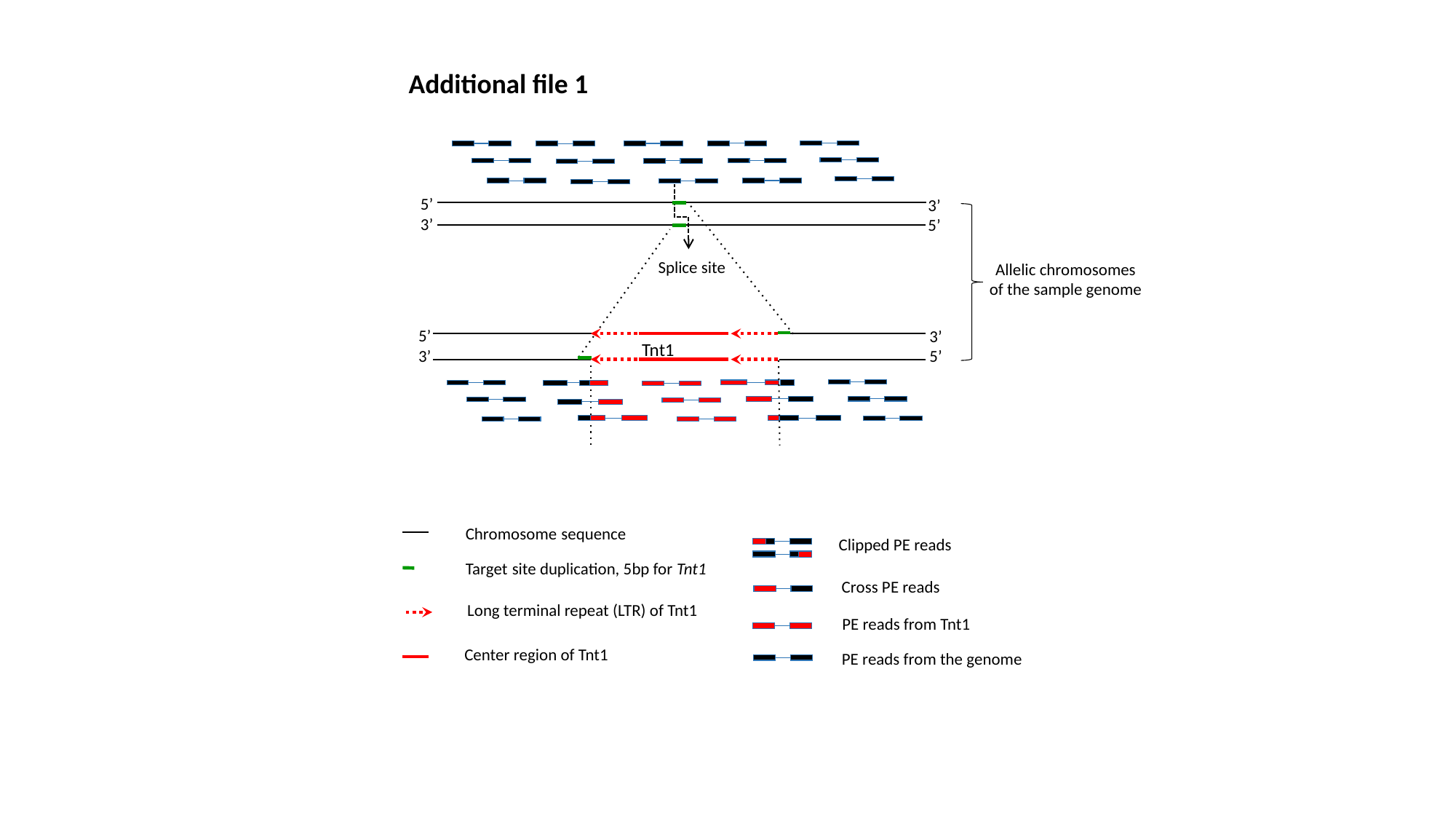

Additional file 1
Tnt1
5’
3’
3’
5’
Splice site
Allelic chromosomes of the sample genome
5’
3’
3’
5’
Chromosome sequence
Target site duplication, 5bp for Tnt1
Long terminal repeat (LTR) of Tnt1
Center region of Tnt1
Clipped PE reads
Cross PE reads
PE reads from Tnt1
PE reads from the genome
