# Supplementary material for: ITIS, a bioinformatics tool for accurate identification of transposon insertion sites using next-generation sequencing data
Source: BMC Bioinformatics. 2015 Mar 5;16(1):72. doi: 10.1186/s12859-015-0507-2 (PMC4351942; doi:10.1186/s12859-015-0507-2)
Supplement: Additional file 3: — Results of PCR amplifications to verify the presence of Tnt1 insertions uniquely identified by TEMP algorithm. Reverse and forward primers were designed on the flanking sequences of the putative insertion sites. In combination with primers designed on both sides of the Tnt1 sequences (5' and 3'), we did not observe any amplification, which suggest that these putative insertions identified by TEMP are false positives. Flanking sequences identified by TEMP are also provided. [file 12859_2015_507_MOESM3_ESM.pptx]

## Slide 1
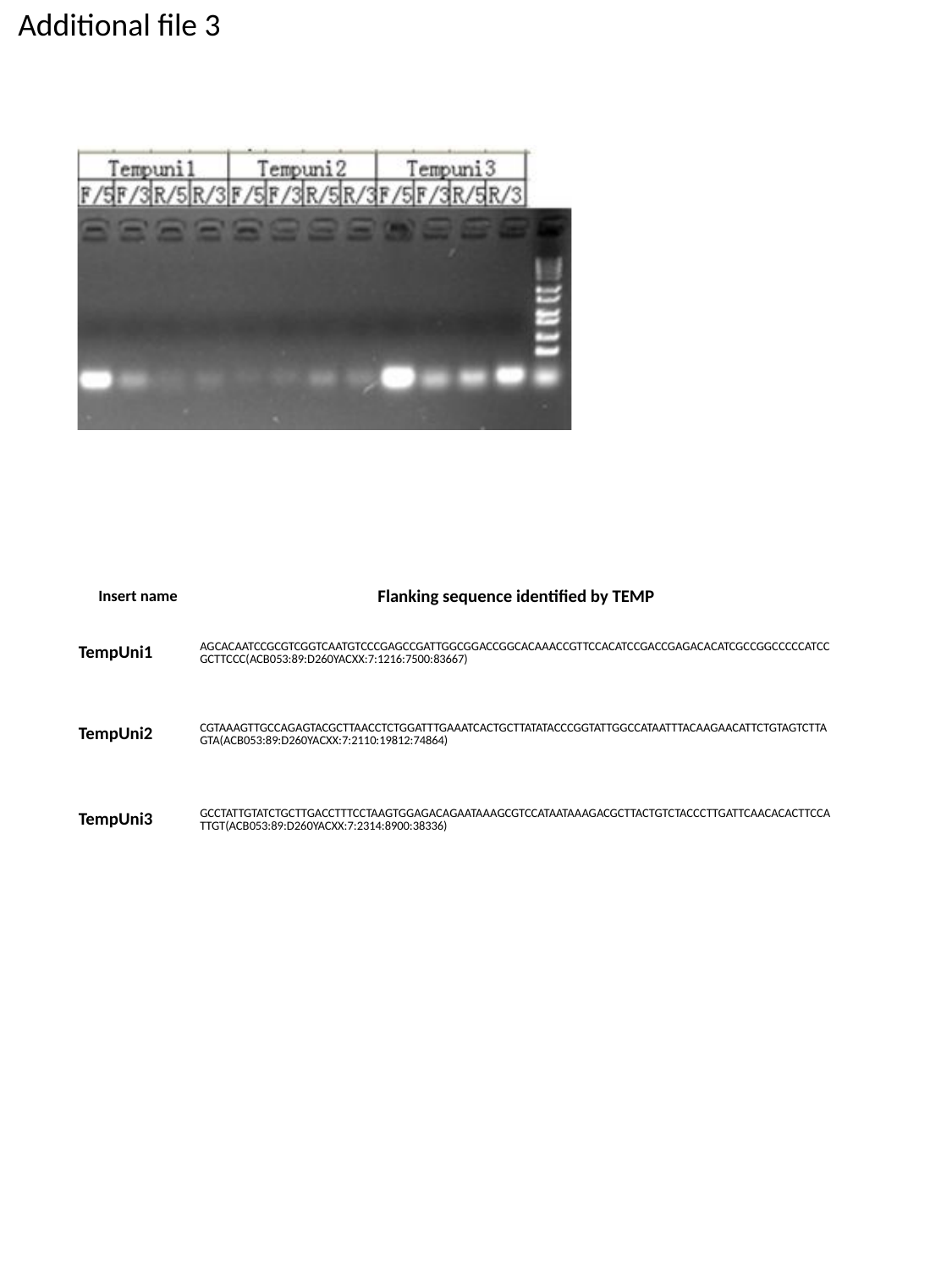

Additional file 3
| Insert name | Flanking sequence identified by TEMP |
| --- | --- |
| TempUni1 | AGCACAATCCGCGTCGGTCAATGTCCCGAGCCGATTGGCGGACCGGCACAAACCGTTCCACATCCGACCGAGACACATCGCCGGCCCCCATCCGCTTCCC(ACB053:89:D260YACXX:7:1216:7500:83667) |
| TempUni2 | CGTAAAGTTGCCAGAGTACGCTTAACCTCTGGATTTGAAATCACTGCTTATATACCCGGTATTGGCCATAATTTACAAGAACATTCTGTAGTCTTAGTA(ACB053:89:D260YACXX:7:2110:19812:74864) |
| TempUni3 | GCCTATTGTATCTGCTTGACCTTTCCTAAGTGGAGACAGAATAAAGCGTCCATAATAAAGACGCTTACTGTCTACCCTTGATTCAACACACTTCCATTGT(ACB053:89:D260YACXX:7:2314:8900:38336) |
