# Supplementary figures and images for: ITIS, a bioinformatics tool for accurate identification of transposon insertion sites using next-generation sequencing data
Source: BMC Bioinformatics. 2015 Mar 5;16(1):72. doi: 10.1186/s12859-015-0507-2 (PMC4351942; doi:10.1186/s12859-015-0507-2)

## Slide 1
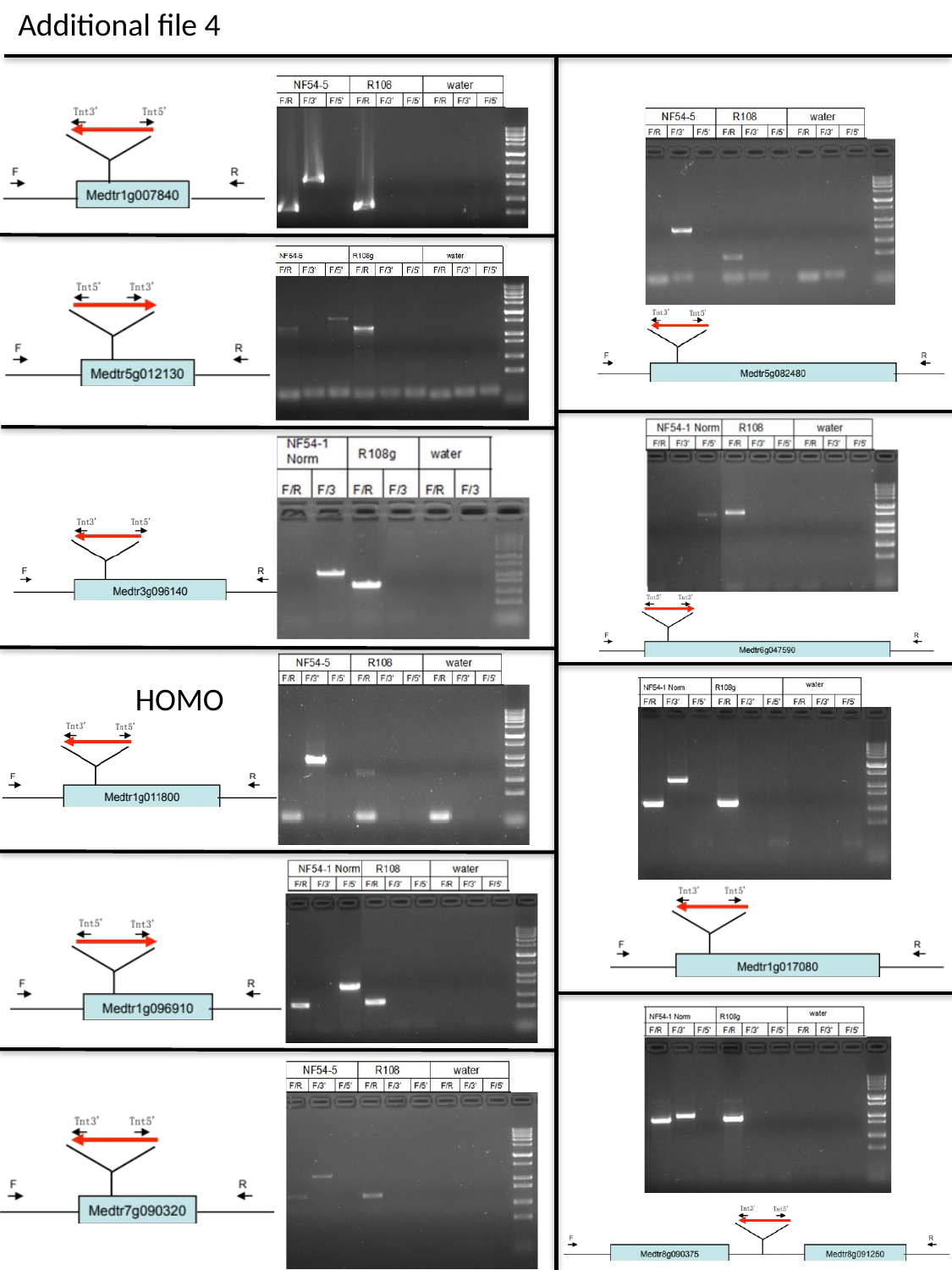

Additional file 4
HOMO

Supplement: Additional file 4: — Results of PCR amplifications to check presence and orientation of Tnt1 insertions identified using ITIS algorithm and not present in the M. truncatula mutant database. Reverse and forward primers were designed on the genomic sequences flanking the putative insertions. In combination with primers designed on both sides of the Tnt1 sequences (5' and 3'), we validated the presence of all tested insertions. According to the combination of primers, we confirmed the orientation of the insertions revealed by ITIS. [file 12859_2015_507_MOESM4_ESM.pptx]
